# Intra-oral low level laser therapy in chronic maxillary sinusitis: A new and effective recommended technique

**DOI:** 10.4317/jced.52282

**Published:** 2015-12-01

**Authors:** Hamed Mortazavi, Hamidreza Khalighi, Ali Goljanian, Robab Noormohammadi, Saeed Mojahedi, Siamak Sabour

**Affiliations:** 1 Associate Professor of Department of Oral Medicine, Dental School, Shahid Beheshti University of Medical Sciences, Tehran, Iran; 2 Assistant Professor of Department of Oral Medicine, Dental School, Shahid Beheshti University of Medical Sciences, Tehran, Iran; 3 Assistant Professor of Department of Otolaryngology, Medical School, Shahid Beheshti University of Medical Sciences, Tehran, Iran; 4 Assistant Professor of Department of Oral Medicine, Dental School, Zanjan University of Medical Sciences, Zanjan, Iran; 5 Associate Professor of Department of Laser, Dental School, Shahid Beheshti, University of Medical Sciences, Tehran, Iran; 6 Associate Professor of Department of Clinical Epidemiology, Dental School, Shahid Beheshti University of Medical Sciences, Tehran, Iran

## Abstract

**Background:**

Chronic sinusitis is one of the most common chronic diseases involving different age groups. Because the nature and etiology of chronic sinusitis are not completely known, there is not any standard treatment for this disease. It has been suggested that low-level laser can be used in treating chronic sinusitis but there are limited studies about its usage. In this research, intra-oral radiation of low-level laser has been described and implemented for the first time. Suggested hypotheses about the efficacy of this type of radiation (intra-oral) in treating chronic maxillary sinusitis includes this fact that the depth of maxilla’s vestibule is also the floor of maxillary sinus and sinus discharges collect in this area because of gravity effect. Therefore, with considering suitable radiation angle, this area gets the most benefits of laser’s anti-inflammatory effects.

**Material and Methods:**

In this study, 20 patients with chronic maxillary sinusitis were included. They were assessed before and after treatment. Treatment plan was performed in 8 sessions every other days using low-level diode laser with 810 nm. Snot-22 questionnaire and rhinomanometry were used for evaluating patients. Changes of signs and symptoms were recorded in questionnaire every session and 6 months after treatment. Friedman and Wilcoxon tests were used for data analyses. In this study, *P* value < 0.05 was considered statistically significant.

**Results:**

All variables and all symptoms of patients were improved using intra-oral low-level laser and this improvement was statistically significant (*P* value<0.05). There was also significant decrease in nasal airway resistance and significant increase in air flow (*P* value<0.05). Six month after treatment completion, there was no significant difference between the results of completion and the results of 8th treatment session (*P* value< 0.05).

**Conclusions:**

Using intra-oral low-level laser is a suitable way to treat patients with chronic maxillary sinusitis.

** Key words:**Chronic sinusitis, maxillary sinusitis, low-level laser.

## Introduction

Chronic sinusitis is one of the most common chronic diseases involving different age groups. People affected have lower life quality compared to the people affected by congestive heart failure, chronic obstructive pulmonary disease and chronic low back pain (1,2). Because the nature and etiology of chronic sinusitis are not completely known, there is not any standard treatment for this disease. Although, there are different drug therapy for draining sinus discharges, decreasing mucosal edema and increasing sinus ventilation and normalizing ciliary function (1-3). There are evidence supporting this hypothesis that inflammation is the major etiologic factor in chronic sinusitis and although using antibiotics and corticosteroids is routine today but the focus is on local therapies, increasing available drug in nasal cavity (nasal delivery) and new anti-inflammatory drugs (3). It must be noted that chronic nature of this disease has influences the treatment because chronic nature needs long term treatment. Researchers have suggested local treatment because of different adverse effects of long term using drugs (3). Nowadays, it is proved that bacterial biofilm has a great role in pathogenesis of chronic sinusitis and in other hand; systemic antibiotics are not mostly effective in treatment infections due to bacterial biofilms. In the cases that there is not anatomical obstruction, functional endoscopic surgeries are not superior to drug therapies. Therefore, because of these problems in treating chronic sinusitis, researchers and clinicians suggested new modalities such as ultrasound therapy and laser therapy (4,5). In laser therapy, extreme radiation with distinct dose irradiates to the surface and exerts its therapeutic effects (anti-inflammatory and anti bacterial effects) with specific mechanisms. Review studies have shown that anti inflammatory effects of low-level lasers are the same as anti inflammatory effects of NSAIDs (5). There are limited studies about therapeutic effects of low-level laser in treating chronic sinusitis (5,6-8). Thus, this is the first research with the aim of evaluating therapeutic effects of intra-oral low-level laser in chronic maxillary sinusitis. In evaluating therapeutic outcomes, it is better considering subjective and objective factors simultaneously (1). According to high cost of some imaging modalities as CT scan in one hand, and non accordance of patients’ symptoms with results of imaging modalities in other hand (9) and also according to the fact that the most common (81-95%) and one of the most important clinical symptoms is nasal obstruction (10,11); researchers of this study begun using rhinomanometry which help them evaluating clinical symptoms objectively and with lower expenditure. Considering all these points, we with the aim of treating patients with chronic maxillary sinusitis and improving their quality of life, tried to answer this question that whether treating with intra-oral low-level laser would treat the patients or not?

## Material and Methods

This study is experimental (interventional) study and performed in before/after manner on 20 patients with chronic sinusitis in Dental school, Shaheed Beheshti University of medical sciences, Tehran, Iran, between 2012-2014. Objectives of the study were explained to patients and written consent was obtained from each of them before performing it. This study was registered in ClinicalTrials.gov with NCT02124538. Physical examinationof all patients were performed by otolaryngologist in Taleqani hospital. Patients’ information was recorded based upon information forms. Symptoms improvement evaluated in two sections: 1- subjective; which was based on patients’ answers in questionnaires. SNOT-22 questionnaire was used in this study, which was validated in 2009, and is recommended in clinical evaluations (12,13). There are 22 questions in this questionnaire including: Need to blow nose, Sneezing, Runny nose, Cough, Post nasal discharge, Thick nasal discharge, Ear fullness, Dizziness, Ear pain, Facial pain/pressure, Difficulty falling asleep, Waking up at night, Lack of a good night’s sleep, Waking up tired, Fatigue, Low performance, Reduced concentration, Frustrated/restless, Sad, Embarrassed, Sense of taste/smell, Blockage/congestion of nose. All these symptoms were assessed in six grades ranging no problem to having severe problems. 2- Objective; which was performed by otolaryngologist using rhinomanoetry test (Rhinomanometry: Ecleris, Rhinosoft, Argentina). In this test, resistance and air flow were measured simultaneously in nasal cavity. Patients should have inclusion criteria: 1- affected by chronic sinusitis: a chronic inflammatory process affecting paranasal sinuses and nasal mucosa, lasting at least 12 weeks and the patient must have two major clinical symptoms or one major and two minor clinical symptoms. 2- Being healthy 3- Not being pregnant or in breast feeding state. 4- Did not have sinus surgery, nasal septum deviation, and nasal polyp. 5- Being cooperative in research. If the patient did not have any of these inclusion criteria, he/she excluded from study. After confirming the disease and recording rhinomanometry curve in the forms and also filling SNOT22 questionnaire for the first time, the patient had been irradiated with low-level laser (Dr. Smile, low level, LAMBDA SpA (Company), France). Intra-oral laser radiation in vestibule depth from canine apical zone to first molar apical zone (floor of the maxillary sinus was performed with 45 degree and without pressure in 10 point with 3 mm distances. This was performed by one person under supervision of laser specialist; then bias probability was decreased. In this method, laser radiation with 810nm and 0.1, 0.4, 0.3, 0.2 W by single probe is irradiated from buccal side. Irradiation was begun every other day with the highest power -0.4W- and ended in lowest power -0.1W- and this cycle repeated two times. (The first day, 0.4 W; second day, rest; third day, 0.3 W; forth day, rest; fifth day, 0.2 W; sixth day, rest; and finally seventh day, 0.1 W). Every session last 40 minutes including resting time (activity time of machine was 20 minutes). Depending on different powers in different days, total dose of energy was 4-7 Joules in every session. There were 8 sessions of irradiation (6). In the end of every session, questionnaires were filled again by patients. In the last session, otolaryngologist again assessed the patients with physical examination and rhinomanometry. Then, the data collected by these questionnaires and also the results of rhinomanometry had been statistically analyzed. Six months after the last treatment, we call patients and the questionnaires were filled again based on their opinion. The results of these calls were also analyzed.

-Data analysis:

Data were analyzed using SPSS software version 18.0. Quantitative and qualitative variables were describes by means and standard deviation and number and percentage. We used Friedman and Wilcoxon tests for data analysis. *P* value < 0.05 was determined as significance level.

## Results

This study included 20 patients (12 females and 8 males) with chronic maxillary sinusitis. In study process, one patient was excluded because of non cooperation. Average age of the 19 patients was 42 (42±16).

The results of SNOT22 questionnaire:

The results obtained about 22 clinical symptoms in this questionnaire are summarized in  Table 11,  Table 1 continue.

**Table 1:**
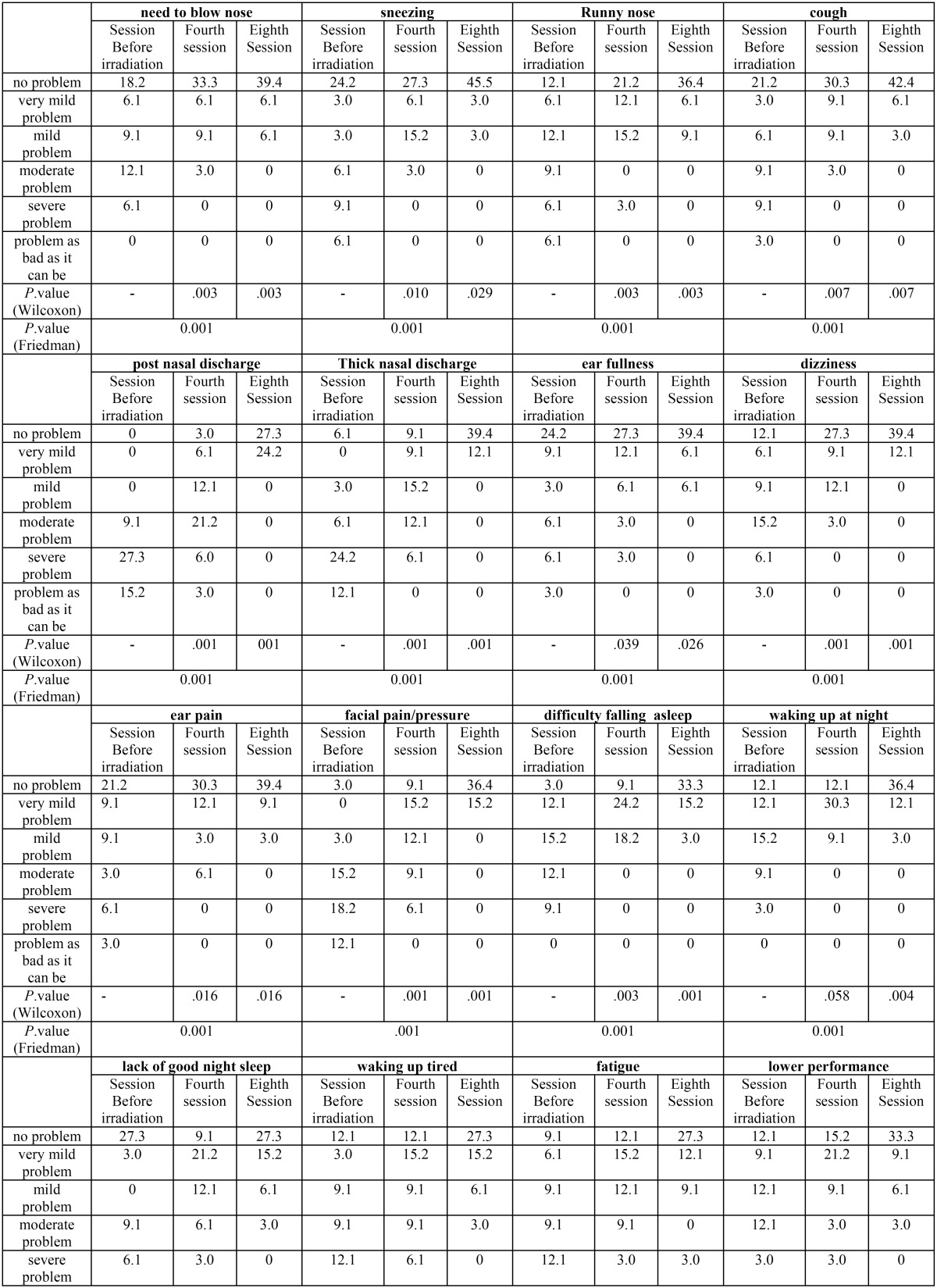
Results from the analysis of variables in snot-22 questionnaire (In Sessions before/middle/end of irradiation).

**Table 1 continue:**
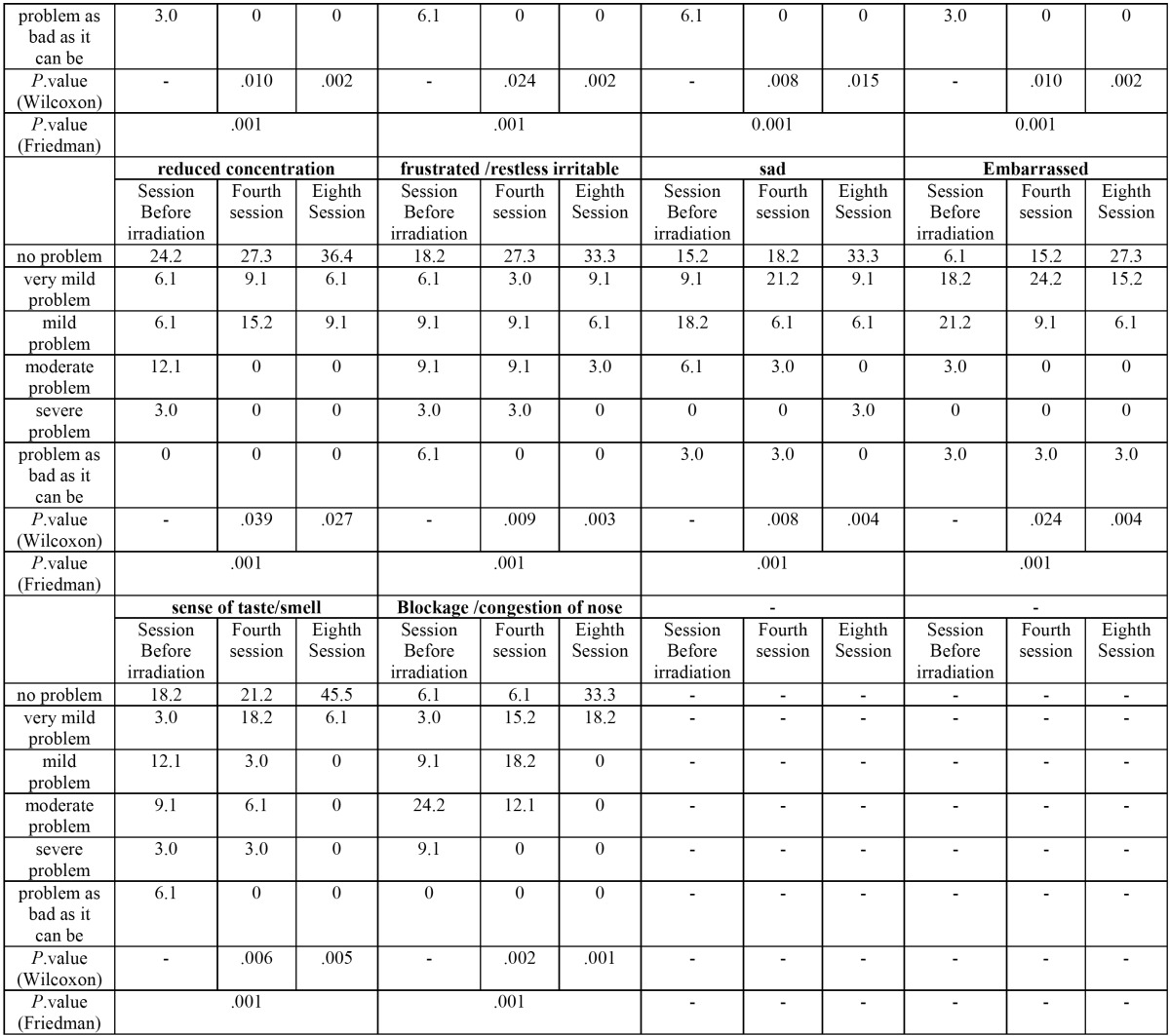
Results from the analysis of variables in snot-22 questionnaire (In Sessions before/middle/end of irradiation).

(1 Due to length of eight sessions of radiation results, Only the results of sessions before of radiation, the fourth and the eighth session are shown in  Table 1).

As seen, percentage distribution of these variables is shown separately for session 1 to session 8.

-In analysis level, we used Friedman test for evaluating whether there is significant decrease of Snot-22 variables or not. The results show that intra-oral radiation decreased these variables significantly (*P*. value =0.001)

-In quantitative evaluation of 22 clinical symptoms in SNOT22 questionnaires, the average score was 44 for the first session and 9 for the 8th session.

-For evaluating the efficacy of intra-oral laser, we used Wilcoxon Signed Ranks Test to assess improving total air flow in 150 Pascal pressure and in decreasing mean total air flow in 150 Pascal pressure. The results obtained in  Table 2 show that intra-oral method increases significantly inspiration and expiration air flow (*P* value= 0.001) and decreases total air resistance in inspiration and expiration.

**Table 2:**
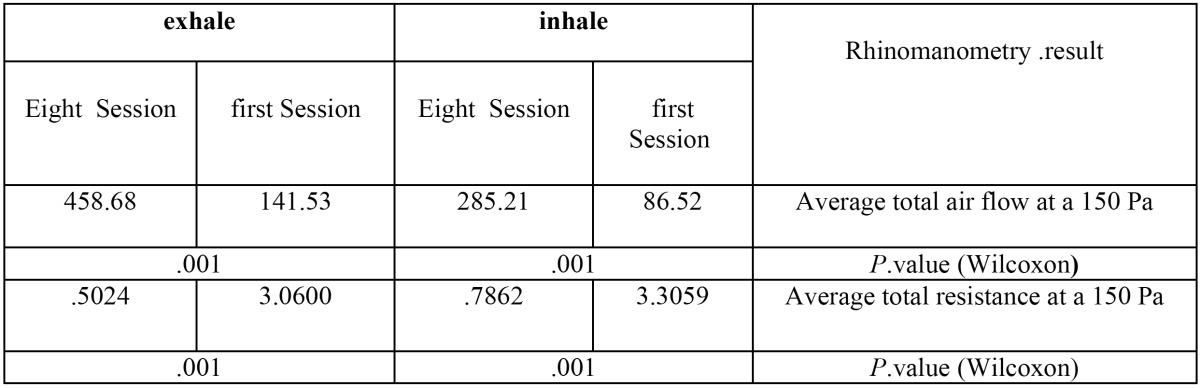
Results of Wilcoxon test on inhale and exhale at 150 Pa.

- Patients’ follow up six months after the last session of intra-oral low-level laser therapy:

Evaluating the data obtained six months after treatment by SNOT22 questionnaires and comprising (by Wilcoxon test) these data with the results obtained in 8th session showed that the patients reported a small reduction in clinical symptoms and there was no significant difference in comparison to 8th session of treatment by Wilcoxon test (*P* value< 0.05).

## Discussion

Intra-oral laser irradiation in treatment of chronic maxillary sinusitis was used in this study for the first time. Then, because of the fact that there is not any similar study, the results of intra-oral radiation cannot be compared with any other study.

It must be noted that in this study low-level diode laser with 810 nm wavelength, output power of 0.1, 0.2, 0.3 and 0.4 W was used to treat chronic maxillary sinusitis. This treatment repeated for 40 minutes in 8 sessions (60 seconds irradiation in 20 points on both sides plus rest time). So, depending on different output powers, received dose for every patient was between 4-7 J/cm2. The cause of using this dose is that doses lower than 4 J/cm2 are not effective on inflamed tissues (7).

Results show that this new treatment method decreased significantly all 22 clinical symptoms included in SNOT22 questionnaires.

It has been shown in adjustment evaluation that there was significant decrease in second treatment session in some variables including: Need to blow nose, sneezing, runny nose, post nasal discharge and thick nasal discharge.

In intra-oral laser irradiation group, prevalence of severe cough and very severe cough decreased to zero in second and third sessions, respectively but significant decrease of its mean was seen in second session.

Intra-oral laser irradiation omitted severe and very severe ear fullness and pain in third and fifth sessions, respectively. But significant decrease of their mean was seen in third session.

Facial pressure/pain was another important variable in case group of study and the most of patients had severe or very severe facial pressure/pain. Severe and very severe cases were omitted in fifth session of treatment. But, significant decrease of its mean was seen in second session.

Sleep related variables (Lack of a good night’s sleep, Difficulty falling asleep, Waking up at night, Waking up tired) decreased significantly in third, third, fifth and second, respectively.

In patients with intra-oral irradiation, fatigue, low performance, Frustrated/restless and sad decreased significantly in third session and the variable, confusion decreased significantly in forth session.

Therefore, we can say that in addition to decreased disease related symptoms means in patients with chronic maxillary sinusitis, psychological symptoms also improved significantly. Sense of taste/smell, blockage /congestion of nose were two major symptoms which were the last items in SNOT22 questionnaire. Their significant decrease was also seen in second session in laser radiation group.

Mean score of SNOT22 questionnaire for all patients were 44. This score was accordant with the score range of patients with chronic sinusitis in other studies. According to the previous studies, mean score of healthy people with the age range of 19-75 was 9.3 and age range of 18-24 was 8.06. After 8 treatment sessions in this study, mean score of intra-oral irradiation group was 9 which was accordant with Gillete study in 2009 (14,15).

In this study, rhinomanometry was used to evaluate laser effects on chronic maxillary sinusitis for the first time. Based on rhinomanometry results, intra-oral irradiation significantly decrease total resistance of nasal cavity and increase total air flow. According to the different studies such as Broms and Suzina, the mean and range of the resistance changes in healthy people were 0.24 (-0.52-0.12) (16,17) which was not accordant to the results of our study. Although,the results of intra-oral irradiation was mostly the same specifically in expiration. In the end of 8th treatment session, airway resistance was 0.7 and 0.5 in inspiration and expiration, respectively.

In resting state, there is at least 200 cm3/sec tidal volume in a standard respiration (12 respirations every minutes), which is accordant to the results of our study (18).

According to the following points, clinical efficacy of intra-oral low-level laser irradiation, which was the most important hypothesis of this study, suggested and proved. Maxilla’s vestibule depth is in fact the floor of maxillary sinus and sinus discharges collected in this zone because of gravity. Thus, using suitable irradiation degree, this zone benefited much from anti inflammatory effects of laser. On the other hand, thickness, skin color, the thickness of underlying muscles and interactions of zygomatic arch which are important in extra-oral irradiation in other studies (5,7,8,19) do not affect the intra-oral laser therapy.

Among 12 studies in this regard, all studies except Moustsen (20) reported positive therapeutically effects of intra-oral low-level laser in the treatment of acute and chronic sinusitis. The results of this study are also in accordance with previous studies.

## Conclusion

Treatment with intra-oral low-level laser significantly improved the clinical signs of SNOT22 and also increased significantly air flow rate and decreased significantly nasal resistance in patients with chronic maxillary sinusitis. Because of different drugs’ side effects, using low-level laser in treatment of chronic maxillary sinusitis is a suitable and conservative choice. Stability of treatment outcomes was desirable according to the results of six month period follow up.
